# *Ex vivo* drug sensitivity profiles of *Plasmodium falciparum* field isolates from Cambodia and Thailand, 2005 to 2010, determined by a histidine-rich protein-2 assay

**DOI:** 10.1186/1475-2875-11-198

**Published:** 2012-06-13

**Authors:** Stuart D Tyner, Chanthap Lon, Youry Se, Delia Bethell, Doung Socheat, Harald Noedl, Darapiseth Sea, Wichai Satimai, Kurt Schaecher, Wiriya Rutvisuttinunt, Mark M Fukuda, Suwanna Chaorattanakawee, Kritsanai Yingyuen, Siratchana Sundrakes, Panjaporn Chaichana, Piyaporn Saingam, Nillawan Buathong, Sabaithip Sriwichai, Soklyda Chann, Ans Timmermans, David L Saunders, Douglas S Walsh

**Affiliations:** 1 Department of Immunology and Medicine, US Army Medical Corps, Armed Forces Research Institute of Medical Sciences (USAMC-AFRIMS), Bangkok, Thailand; 2 The National Center for Parasitology, Entomology and Malaria Control (CNM), Phnom Penh, Cambodia; 3 Institute of Specific Prophylaxis and Tropical Medicine, Medical University of Vienna, Kinderspitalgasse 15, Vienna, A-1090, Austria; 4 Bureau of Vector-Borne and Zoonotic Diseases, Thailand Ministry of Public Health, Nonthaburi, Thailand

**Keywords:** Cambodia, malaria, *Plasmodium falciparum*, HRP-2, Anti-malarial drugs, Drug resistance

## Abstract

**Background:**

*In vitro* drug susceptibility assay of *Plasmodium falciparum* field isolates processed “immediate *ex vivo*” (IEV), without culture adaption, and tested using histidine-rich protein-2 (HRP-2) detection as an assay, is an expedient way to track drug resistance.

**Methods:**

From 2005 to 2010, a HRP-2 *in vitro* assay assessed 451 *P. falciparum* field isolates obtained from subjects with malaria in western and northern Cambodia, and eastern Thailand, processed IEV, for 50% inhibitory concentrations (IC_50_) against seven anti-malarial drugs, including artesunate (AS), dihydroartemisinin (DHA), and piperaquine.

**Results:**

In western Cambodia, from 2006 to 2010, geometric mean (GM) IC_50_ values for chloroquine, mefloquine, quinine, AS, DHA, and lumefantrine increased. In northern Cambodia, from 2009–2010, GM IC_50_ values for most drugs approximated the highest western Cambodia GM IC_50_ values in 2009 or 2010.

**Conclusions:**

Western Cambodia is associated with sustained reductions in anti-malarial drug susceptibility, including the artemisinins, with possible emergence, or spread, to northern Cambodia. This potential public health crisis supports continued *in vitro* drug IC_50_ monitoring of *P. falciparum* isolates at key locations in the region.

## Background

Since the 1980s, measuring *in vitro* drug 50% inhibitory concentrations (IC_50_) against *P. falciparum* field isolates has been useful in tracking clinical drug susceptibility patterns [1-3]. In Southeast Asia, especially along the Thailand-Cambodia border, changes in drug susceptibility often emerge first, with worldwide implications, underscoring the region’s importance as a sentinel site for anti-malarial drug resistance [3].

In 2003, artesunate (AS) + mefloquine (MQ) was implemented as the first-line artemisinin-combination therapy (ACT) for falciparum malaria in Cambodia. By 2005, subjects with falciparum malaria along Thai-Cambodia border treated with oral artesunate were showing longer parasite clearance times, suggesting the emergence of reduced susceptibility to artemisinins, as well as to the partner drug [4].

The non-radioisotope histidine-rich protein-2 (HRP-2) ELISA, a relatively sensitive assay, which reliably depicts drug IC_50_ values for *P. falciparum* isolates, reduces obstacles for conducting assays in remote settings [5,6]. HRP-2 assays are expedient, safer and less costly than radioisotope assays. HRP-2 assay, when field deployed proximal to *P. falciparum* collection sites, allows for “immediate *ex vivo*” (IEV) field isolate processing [7]. IEV, by avoiding cryopreservation and culture-adaptation before IC_50_ determination, may reduce clonal selection, better preserving parasite subpopulations with variable drug susceptibility profiles, the latter likely present in a smaller proportion than wild-type drug susceptible parasites.

Here, to characterize recent geographical and temporal trends in western and northern Cambodia, and eastern Thailand, including years when reduced artemisinin susceptibility was first described, an HRP-2 assay, with IEV field isolate processing was used to determine IC_50_ values of *P. falciparum* field isolates obtained from 2005 to 2010.

## Methods

### Protocol, sites and subjects

**Figure 1:**
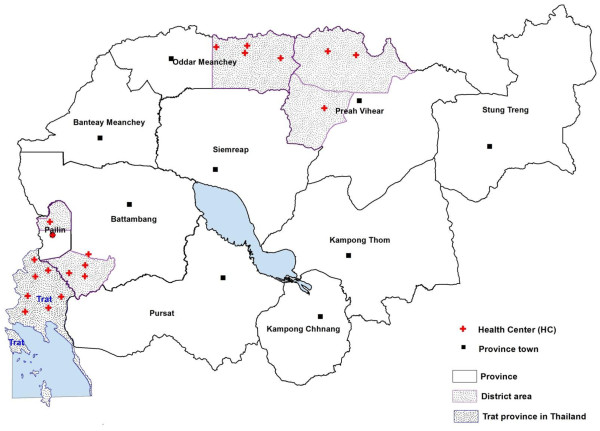
**Map depicting provinces in Cambodia and Thailand (shaded) where*P. falciparum*isolates were obtained.**

All studies were approved by 1 or more ethical review boards which included Cambodian National Ethics Committee for Health Research (NECHR), Ethical Review Committee for Research in Human Subjects, Thailand Ministry of Public Health (MoPH), Walter Reed Army Institute of Research (WRAIR) Institutional Review Board and World Health Organization (WHO) Ethical Review Committee (protocol numbers: WRAIR 973, WRAIR 1327, WRAIR 1296, WRAIR 1396, WRAIR 1576, and WRAIR 1737). All protocols complied with International Conference on Harmonization Good Clinical Practice (ICH-GCP) guidelines. Enrollment centers were located in western and northern Cambodia (Pailin, Battambang, Oddar Meanchey and Preah Vihear Provinces (Figure 1), and Trat, eastern Thailand. All sites were considered low transmission, malaria endemic regions at the time of sample collection [8,9].

Among six studies involved in subject recruitment, summarized in Table 1, subjects of five to 70 years of age with smear-confirmed, uncomplicated *P. falciparum* malaria were eligible to participate. Written informed consent was obtained from adult subjects, or from legal guardians with assent from minors. Subjects who gave a history of anti-malarial drug use within the previous 7–30 days, were pregnant or nursing, or who had signs suggestive of severe malaria, were excluded from participation (Table 1). Treatment following malaria diagnosis was according to national standards of care at the respective times, or involved randomized allocation to an artemisinin containing regimen as part of an interventional clinical trial.

**Table 1:** **Studies providing*P. falciparum*isolates for HRP-2 IC**_**50**_**drug assays**

**Study**	**Years of enrolment**	**Location**	**Relevant inclusion criteria**
		**Province, Country**	
1	2005-07	Trat, Thailand^1^	> 19 years old, any asexual *P. falciparum* parasitaemia
2	2006-07	Trat, Thailand^1^	5-70 years old, any asexual *P. falciparum* parasitaemia
3	2006-07	Western Cambodia^2^:	18-65 years old, asexual *P. falciparum* parasitaemia, > 100 and < 100,000/μL
		Battambang	
4	2008-09	Western Cambodia^2^:	18-65 years old, asexual *P. falciparum* parasitaemia, > 1000 and < 200,000/μL
		Battambang	
5	2009-10	Western Cambodia^2^:	≥ 13 years old, any asexual *P. falciparum* parasitaemia
		Battambang, Pailin	
		Northern Cambodia^3^:	
		Oddar Mean Chey	
		Preah Vihear	
6	2010-11	Northern Cambodia^3^:	18-65 years old, any asexual *P. falciparum* parasitaemia
		Oddar Mean Chey	
		Preah Vihear	

^1^ Thailand Ministry of Public Health Clinics in Borai, Khaosaming and Muang districts.

^2^ Western Cambodia: Pailin (Pailin Hospital) and Battambang Provinces (Tasanh, Samlot, Chanlong Kuoy and Kampong Lpov health centers);

^3^ Northern Cambodia: Oddar Mean Chey (Anlong Veng, Trapang Tav, Trapang Prey, Trapang Prasat and Sraem health centers), and Preah Vihear Provinces.

### Malaria microscopy

Microscopic examination of Giemsa-stained peripheral blood smears was performed by two microscopists for each subject (200 high-powered fields [HPF] per reader) to determine parasitaemia, and presence/absence of a second malaria species. Microscopists conducted all reads in the thick smear unless there were > 500 parasites/200 WBCs, in which case they switched to the thin smear. Discrepancies between microscopists were resolved in real-time by a third reader (reviewing an additional 200 HPFs), who determined the final result. Parasite density was calculated as the number of parasites/200 WBC’s in the thick smear or the number of parasites/5000 RBC’s in the thin smear. A geometric mean (GM) of the two microscopist’s results was used as the final parasitaemia for each subject. Time zero peripheral blood specimens from WRAIR 1396, 1576, and 1737 were also confirmed by species-specific real-time PCR (RT-PCR) targeting the 18 s rRNA gene. Specimens with a microscopically or RT-PCR confirmed malaria co-infection at time zero, *Plasmodium vivax* or other malaria species, were excluded from analysis. Subject parasite density ranges at study enrolment are shown in Table 1.

### Sample collection and preparation

Enrolled subjects provided up to 8 ml of venous blood in sodium heparin tubes for the HRP-2 *ex vivo* IC_50_ assay. In all cases, *P. falciparum* isolates from submitting study locations were plated directly onto malaria drug coated plates without prior culture adaptation [5]. All applications of falciparum isolates to drug coated plate occurred at field sites or in central laboratories located at Battambang Referral Hospital (BRH) or at Anlong Veng Health Center (AVHC). Samples analyzed at BRH or AVHC were refrigerated during transportation (2 - 8°C) and plated < 6 hours after being drawn from the subject.

### Drugs and plate coating

Seven anti-malarial drugs for IC_50_ testing were used: chloroquine diphosphate (CQ), mefloquine hydrochloride (MQ), quinine sulfate hydrate (QN) artesunate (AS), dihydroartemisinin (DHA), lumefantrine (LUM) and piperaquine (PPQ). CQ, MQ, QN and PPQ were provided as salts, AS, DHA, and LUM as bases. All drug stocks were prepared using a weight/volume convention with salt or base formulation weights used to calculate nM IC_50_ values. Test drugs were provided by Walter Reed Army Institute of Research (Silver Spring, Maryland, USA).

To prepare test drugs, stock drug solutions at 1 mg/mL were prepared in 70% ethanol for DHA, AS, MQ, QN, and CQ or a solution of 100% ethanol : linoleic acid : Tween 80 (1:1:1) for LUM and 0.5% lactic acid in distilled water for PPQ. Further drug dilutions were performed in distilled water for DHA, AS, MQ, QN, CQ, and PPQ or 70% ethanol for LUM to the desired starting concentration, followed by serial 3-fold dilutions to generate 7 concentrations for IC_50_ testing. The last row of the drug plate contained no drugs. Dried drug coated plates were prepared as described [5,6,10].

The concentration ranges (ng/ml), from highest to lowest, and molecular weights (g/mole) used to convert to nM were, respectively: CQ (2000 to 2.74; 515.92), MQ (200 to 0.274; 414.78), QN (1250 to 1.71; 782.97), AS (20 to 0.027; 384.42), DHA (20 to 0.027; 284.35), LUM (50 to 0.07; 528.95) and PPQ (625 to 0.86; 999.56). The drug solutions were prepared and used within one week. Drug solutions were stored at 2 – 8°C and used within 1 week of preparation.

### *Plasmodium falciparum* W2 reference clone and quality control

W2, a well characterized *P. falciparum* laboratory reference clone, recovered from cryopreserved sample and maintained in continuous culture for ≤ 5 months, served as quality control for drug coating plate lots and to assess assay performance within a previously validated acceptable range. W2 also served as a comparator for *P. falciparum* field isolate IC_50_ values. W2 IC_50_ assays against each drug were conducted annually from 2008 to 2010. W2, *in vitro*, is considered CQ “resistant” (CQ-R), and MQ “susceptible” (MQ-S). The W2 GM IC_50_ values of CQ, MQ and QN, especially when assessed simultaneously with *P. falciparum* field isolates, provide context for non-radioisotopic IEV assays [11]. W2 has no defined IC_50_ value discriminative for AS or DHA “resistance”.

Earlier reports describe drug IC_50_ “cut-off” values for “culture-adapted” *P. falciparum* field isolates, once generally considered discriminative for *in vivo* resistance; these include CQ (> 45.5 ng/ml; 85 nM), MQ (> 10 ng/ml; 24 nM), and QN (> 275 ng/ml; 351 nM) [12,13]. These historical cut-off values may provide rough context, primarily for assay reliability, and perhaps for *P. falciparum* field isolate IC_50_ trends, including non-radioisotopic IEV assays [11].

For quality control (QC), conducted from 2008 to 2010, a subset of all drug-plate coating lots was tested using the HRP-2 assay with W2 and an IC_50_ value was derived for each anti-malarial compound before the lot was sent to the field. Lots that performed within previously validated criteria were cleared to be sent to the field. Out of range lots were destroyed. Drug plating consistency and stability between different lots were consistent with previous reports [14]. A subset of each plate-coating lot taken to the field was stored refrigerated (2 - 8°C) for up to 8 weeks, then transported back to the AFRIMS laboratory in Bangkok where QC was performed again with W2, to determine if transport and storage conditions in the field affected IC_50_ analysis.

### HRP-2 IC_50_ drug susceptibility assay

An established HRP-2 IC_50_ drug susceptibility assay was used to assess each *P. falciparum* field isolate, processed immediate *ex vivo*[5,6]. Falciparum isolates were processed within six hours after phlebotomy, including parasitaemia adjustment, as previously described, then placed onto drug coated plates (0 hours) [5,15]. Falciparum isolates within the range of plate parasitaemia (0.2% – 0.5%) were immediately transferred to the drug coated plates as the 0 hr screening sample; samples with a parasitaemia > 0.5% were diluted to within the plate parasitaemia range by adding 50% haematocrit human O + red blood cells in 10% serum RPMI 1640 culture media; and samples with a parasitaemia below 0.2% were plated without adjustment [15].

Samples (~200 μl) from untreated wells were collected every 24 hours for 72 hours, with parasite growth rate interpreted as the difference in HRP-2 production between 0 and 72 hours. Due to the high sensitivity of the HRP-2 assay, dilution of the samples was sometimes required before performing the ELISA to bring the concentration within assay OD range.

### Statistical analysis of IC_50_ values

HRP-2 optical densities (OD) were plotted against drug concentrations and IC_50_ values estimated by nonlinear regression analysis using ICEstimator [16]. In *P. falciparum* field isolates which showed measurable growth, ICEstimator rules were used to determine the concentration-response relationship and IC_50_, i.e., ratio of growth without drug to that with maximum drug, For each drug, IC_50_ values were expressed as geometric means (GM). Statistical significance within and between groups was determined by nonparametric Kruskal-Wallis or Mann–Whitney tests, as indicated (Graph-Pad Prism 4, GraphPad Software, Inc., La Jolla, California, USA).

## Results

### HRP-2 IC_50_ drug assays

Among 590 *P. falciparum* isolates collected, IC_50_ values on 451 (76%) were obtained.

The success rate for all assays was about 75%, including 78% with a parasitaemia > 0.5% and 61% with a parasitaemia ≤ 0.5.

**Figure 2:**
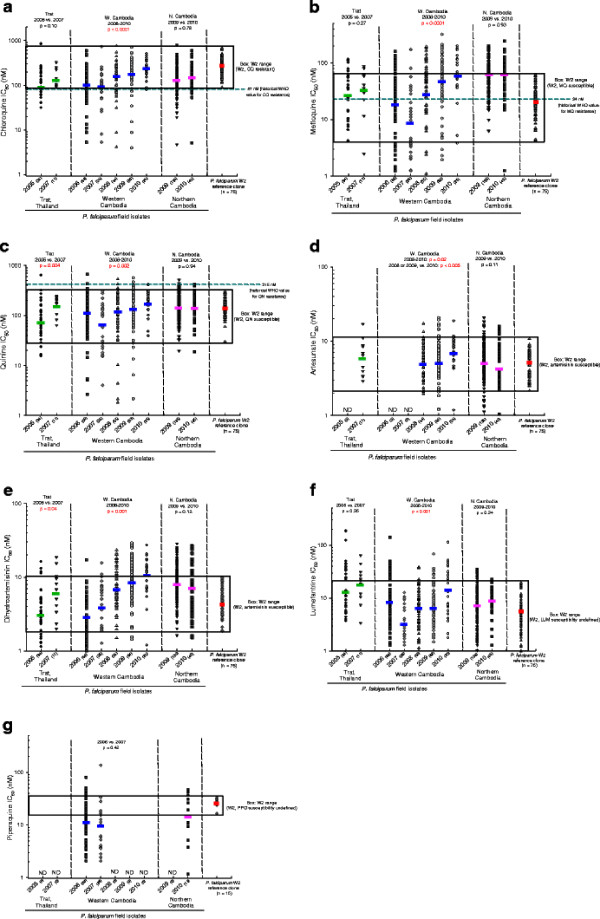
**(a-g):*In vitro*HRP-2,*P. falciparum*field isolate IC**_**50**_**values (nM), determined by HRP-2 against 7 drugs shown as a scatter plots, with geometric mean (GM; solid color bars), by year and region.** Sample numbers, in parentheses, are next to each year. P values at top of graphs determined by Mann–Whitney (2 groups) or Kruskal-Wallis. (> 2 groups) tests; p < 0.05 in red. *P. falciparum* W2 reference clone values for each drug shown at far right (GM, solid red bars); boxes denote ranges. Dashed horizontal lines (green) on CQ, MQ and QN graphs represent approximate historical. IC_50_ values, denoting clinical “resistance”. nM = nanomolar. ND = not done.

Figure 2 (a-g) shows individual and geometric mean (GM) IC_50_ values for IEV *P. falciparum* field isolates, by location and year, and W2 *P. falciparum* reference clone (CQ-R, MQ-S, QN-S, artemisinin-S) IC_50_ values against each drug. In western Cambodia, assessed annually from 2006 to 2010, geometric mean GM IC_50_ values for CQ, MQ QN, AS, DHA, and LUM increased (p < 0.05). In northern Cambodia, assessed in 2009 and 2010, GM IC_50_ values for most drugs approximated the highest western Cambodia GM IC_50_ values in 2009 or 2010. In Thailand, assessed in 2005 and 2007, GM IC_50_ values increased for QN and DHA (p < 0.05).

## Discussion

Among *P. falciparum* field isolates obtained in western Cambodia from 2005 to 2010, steady increases were observed for GM IC_50_ values measured by a HRP-2 *in vitro* assay against a range of anti-malarial drugs, including AS and DHA. Moreover, in northern Cambodia, assessed in 2009 and 2010, most GM IC_50_ values approximated those in western Cambodia, during the same period. This supports the notion that western Cambodia is associated with sustained and likely progressive reductions in anti-malarial drug susceptibility, with possible spread to northern Cambodia.

As the same HRP-2 IEV assay was used for all *P. falciparum* field isolates, increasing GM IC_50_ values for AS and DHA may reflect possible emerging artemisinin resistance, first described in subjects in western Cambodia in 2005 [7,17,18]. Statistically significant increases in GM IC_50_ values for DHA in Trat Province, Thailand, adjacent to the Cambodian border, from 2005 to 2007, coincided with declining efficacy of AS + MQ in Thailand [19]. These observations may reflect a > 30 year history of artemisinin use in this region.

In western Cambodia, a steady increase in GM IC_50_ values for CQ from 2006 to 2010 was observed. This may reflect continued drug pressure due to its use for *P. vivax* blood stage treatment, as well as widespread unregulated availability. This is in contrast to the situation in parts of Africa, where the introduction of ACTs and withdrawal of CQ use has led to a reduction in mean IC_50_ values from those recorded when CQ was the first-line treatment for *P. falciparum* infections [20,21].

QN showed no remarkable trends in GM IC_50_ values, paralleling continued effectiveness of this 2nd line agent. For LUM, not widely used in Cambodia, increasing GM IC_50_ values in western Cambodia from 2006–2010, in parallel with trends in other drugs, possibly mirrored increases in MQ IC_50_s (discussed below). For PPQ, the clinical relevance of our preliminary *in vitro* IC_50_ data remains unclear. Of note, 15% of subjects in two recent malaria treatment trials in Cambodia had detectable pre-treatment PPQ levels, suggesting increasing drug pressure [22]. Continued *in vivo* and *ex vivo* monitoring of PPQ, the recently adopted first-line partner drug for DHA in Cambodia, is of paramount importance.

In western Cambodia, MQ GM IC_50_ values showed a steady increase from 2006 to 2010, and comparably high GM IC_50_ values were observed in northern Cambodia in 2009–2010; all were well above the proposed WHO historical cut-off indicative of clinical MQ monotherapy “resistance”, paralleling reduced clinical efficacy of MQ in this region [23,24]. The cause of reduced MQ sensitivity (and possibly LUM) is generally accepted to be related to increased *Pfmdr*1 copy number, so the association of increased MQ GM IC_50_s with a recent description of reduced Pfmdr1 copy numbers in western Cambodia from 2005 to 2007 is unclear [25]. The WHO 2010 Global Report suggests this molecular event was due to a switch in treatment policy from MQ-AS to DHA-PPQ [4]; if so, a continued rise in MQ mean IC_50_ values to 2010 is potentially concerning. As before, unregulated availability of antimalarial medications during that period may have been a contributing factor. Notably, MQ-AS still appears to retain high efficacy despite reduced *in vitro* sensitivity to both drugs, shown most recently in a trial comparing mefloquine-artesunate with pyronaridine-artesunate [26].

For northern Cambodia, little is known about IC_50_ malaria drug susceptibility [27]. Relatively high GM IC_50_ values in northern Cambodia in 2009 and 2010 are supported by antecedent GM IC_50_ values in eastern Thailand and western Cambodia [27-29]. In Thailand, GM IC_50_ values for CQ, MQ, QN and LUM increased from 2005 to 2007, an increase mirrored in nearby western Cambodia, starting in 2006. QN GM IC_50_ values remained below the WHO historic cut-off of 315 nM denoting "resistance", whereas CQ and MQ GM IC_50_ values rose above WHO historic cut-off values, and DHA showed marked increases in both countries. These trends followed drug policies during the survey period, with AS-MQ the first-line regimen for *P. falciparum*, and CQ as the first-line agent for blood stage *P. vivax* in both Thailand and Cambodia.

In western Cambodia, measured annually 2006–2010, statistically significant, relatively steady increases in GM IC_50_ values for CQ, MQ, QN, AS, DHA and LUM were noted. However, GM IC_50_ values dropped in 2007, versus 2006, but then steadily increased from 2008 to 2010. It is unclear why GM IC_50_ decreases occurred in 2007 only. Review of data from 2007 showed a smaller sample size (n = 26) compared to other years, although the location of sampled sites was unchanged. Interestingly Lim *et al.* observed a similar dip in IC_50_ values for a range of antimalarial drugs in 2006, and attributed the observation to sampling bias since most samples that year were collected from a single, new field site; the data was included in the report for completeness and so that future comparisons could be made [27]. This perhaps illustrates the importance considering factors such as sampling bias when interpreting antimalarial drug IC_50_ surveys.

HRP-2 assays with IEV isolate processing conducted at field laboratories, which are simpler techniques than radioisotopic assays and culture-adaptation, may also reduce clonal selection and better preserve sub-populations of susceptible and potentially drug resistant parasites [11]. In this survey about 75% of HRP-2 IEV assays were successful, and W2 clone IC_50_ values for CQ, MQ, QN and AS were within expected ranges, providing context for falciparum isolate IC_50_ values. This bodes well for field based HRP-2 IEV assays. Moreover, GM IC_50_ values reported here generally paralleled other published data from eastern Thailand and Cambodia, which assayed *P. falciparum* isolates by ^3^ H-hypoxanthine uptake [28,30]. For example, a comparison of western Cambodia GM IC_50_ ranges from 2001 to 2007 [27], with these results were, respectively (nM): CQ (131–237 vs. 96–242), MQ (12–57 vs. 7–58), QN (94–302 vs. 53–163), and AS (0.6-1.8 vs.5.1-6.8). The higher AS GM IC_50_ range reported here could reflect continuing emergence of artemisinin resistance, paralleling GM IC_50_ increases for AS and DHA from 2008 to 2010. Alternatively, it might reflect methodological differences, which affect drug or parasite behaviour, illustrating the importance of inter-assay consistency.

Trends in IC_50_ values, coupled with molecular marker assays, help track emergence and spread of anti-malarial drug resistance. For the artemisinins, although associations between validated resistance markers, treatment outcome and IC_50_ susceptibility remain elusive [18,31]., in part because the clinical definition of artemisinin “resistance” is unclear; the converse is not true for partner drugs, such as MQ and CQ, which have established thresholds of resistance and validated molecular markers. Nonetheless, determining AS and DHA GM IC_50_ values in a substantial number of *P. falciparum* isolates from 2005 to 2010 establishes a baseline which may be useful for future comparisons if evidence of reduced artemisinin susceptibility grows, and especially if the methodology remains consistent.

## Conclusion

Increases in GM IC_50_ values for a range of important anti-malarial drugs in a region of emerging drug resistance underscores the importance of harmonizing methodologies that allow for accurate comparison between geographical locations, and over time, so that potentially important trends in drug susceptibility can be identified.

## Competing interests

The authors declare that they have no competing interests.

## Authors’ contributions

Study designs, oversight, data interpretation, manuscript preparation: CL, ST, DS (Dr Socheat), YS, HN, DB, KS, WR, WS, MF, SC (Dr. Suwanna), AT, DS (Dr Saunders), DW Subject recruitment, interactions: DS (Dr Sea), YS, SC (Ms. Chann), NB, SS (Ms Sabaithip) Conducted experiments, summarized data: ST, KS, WR, SC, KY, SS (Ms Siratchana), PC. All authors read and approved the final manuscript.
